# Enhanced thermoelectric performance in Sb–Br codoped Bi_2_Se_3_ with complex electronic structure and chemical bond softening†

**DOI:** 10.1039/d1ra08726f

**Published:** 2022-01-11

**Authors:** Ju Zhang, Shiqi Zhong, San-Huang Ke

**Affiliations:** MOE Key Laboratory of Microstructured Materials, School of Physics Science and Engineering, Tongji University 1239 Siping Road Shanghai 200092 China shke@tongji.edu.cn

## Abstract

Prior experimental work showed that Bi_2_Se_3_, as a sister compound of the best room-temperature thermoelectric material Bi_2_Te_3_, has remarkably improved thermoelectric performance by Sb–Br codoping. But the relationship between its crystalline structure and thermoelectric properties is still unclear to date. Here, we use first-principles calculations to explore the possible reasons for such improvement. The electronic structures of Bi_2−*x*_Sb_*x*_(Se_1−*y*_Br_*y*_)_3_ (*x* = 0, 1, 2; *y* = 0, 0.08) are systematically investigated. Significant effects of 8% Br codoping in BiSbSe_3_ are found. First, the Br atom acts as an electron donor, thus greatly increasing the carrier concentration. Second, similar to the effect of Sb doping, Br codoping further improves greatly the degeneracy of the conduction band edge, which leads to a remarkably increased density-of-states effective mass without deterioration of the carrier mobility, and simultaneously preserves a large Seebeck coefficient of ∼−254 μV K^−1^ at 800 K. In addition, the Br codoping softens the chemical bonds, which enhances anharmonic scattering and further reduces the lattice thermal conductivity. We predict that the maximum *zT* of BiSb(Se_0.92_Br_0.08_)_3_ at 800 K can reach 0.96 with the carrier concentration of 9.22 × 10^19^ cm^−3^. This study rationalizes a potential strategy to improve the thermoelectric performance of Bi_2_Se_3_-based thermoelectric materials.

## Abstract 2

A detailed description of the charge density difference of BiSb(Se_0.92_Br_0.08_)_3_.

## Graphical abstract

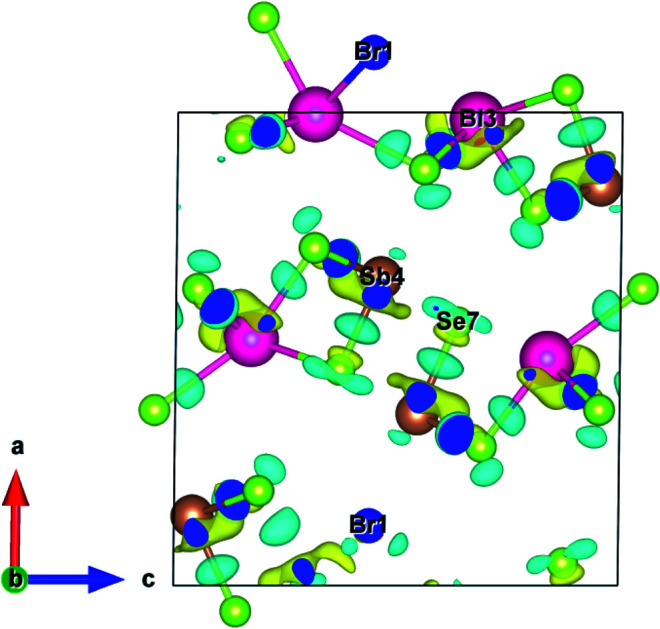

## Introduction

With about two-thirds of the world's produced energy being lost as waste heat,^1^ thermoelectric (TE) materials which can directly convert exhaust heat into usable electricity have been investigated widely as clean and sustainable energy materials.^2^ The TE performance depends on the dimensionless figure of merit *zT* = *S*^2^*σT*/*κ*, where *S*, *σ*, *κ*, and *T* are the Seebeck coefficient, electrical conductivity, total thermal conductivity (including the lattice contribution *κ*_L_ and carrier contribution *κ*_e_), and absolute temperature, respectively.^3–5^ An excellent TE material requires a high value of *zT* simultaneously with a high power factor (PF = *S*^2^*σ*) and a low *κ*.^6,7^ Because of the strong coupling between *S*, *σ*, and *κ*_e_, it is very difficult to enhance *zT* by optimizing only one of these parameters.^8^ To date, decoupling of these electronic parameters by band engineering approaches through increasing the number of effective energy valley and minimizing *κ*_L_ (the only independent parameter) have proved to be effective strategies for enhancing *zT*.^9^

It is well known that the electrical transport properties of a material are dominated by the details of its band structure and scattering mechanism. The optimized electrical transport properties of a thermoelectric material depend on the weighted mobility,^10^
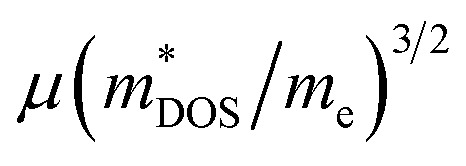
, here *μ*, 
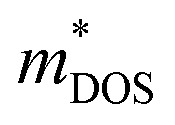
 and *m*_e_ are the mobility of carriers, density-of-states (DOS) effective mass and electron mass, respectively. The DOS effective mass is given as^11^
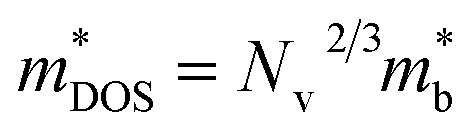
, where *N*_v_ is the band degeneracy and 
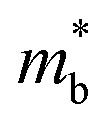
 is the band effective mass. Actually, for the charge carriers predominantly scattered by acoustic phonons, the mobility is expected that 
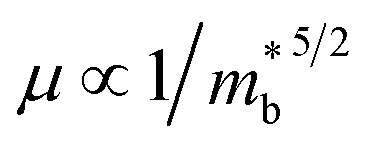
.^12^ Consequently, increasing the 
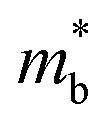
 should be detrimental to the thermoelectric performance. In contrast, the convergence of many charge carrying valleys has virtually no detrimental effects. Therefore, multiple degenerate valleys are generally desired, thanks to the separate pockets of Fermi surface with the same energy, which have the effect of producing large 
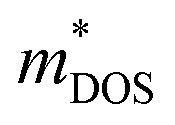
 without explicitly increasing 
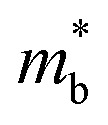
. Besides, materials with soft chemical bonds and anharmonic lattice dynamics would suppress the lattice thermal conductivity.^1,13,14^ However, the combination of all these features has been identified^12,15^ with a large challenge in achieving high-performance thermoelectrics through an avenue simultaneously possessing large band degeneracy and strong anharmonic lattice dynamics, which are highly inter-dependent. As the best commercialized thermoelectric material found so far, Bi_2_Te_3_ has excellent *σ*, *S*, and large *zT* (for both n-type and p-type), and therefore have been widely applied for TE power generation and electronic cooling around room temperature.^16^ However, Te is a scarce element in the crust of the earth and its cost would rise sharply along with large-scale applications. Therefore, Te-free TE materials have attracted great attention.

As an important low-cost thermoelectric material, Bi_2_Se_3_, a sister compound of Bi_2_Te_3_, has also been receiving attention due to the constituent of abundant elements. Specifically, it possesses the same layered structure and the weak inter-layer van der Waals bonding, which makes it easier to be separated into plates.^17^ Smaller plates with more interfaces mean lower *κ*_L_ theoretically, which is very favorable to thermoelectric applications. However, Bi_2_Se_3_ has drawn little attention owing to its relatively poor TE performance with *zT* being ∼0.4.^18^ Many efforts have been focused on optimizing the TE merit value of Bi_2_Se_3_ in recent years. Liu *et al.* demonstrated that the key limitations for the *zT* of Bi_2_Se_3_ are its poor electrical transport properties and high *κ*.^18,19^ With the intrinsically small band gap (*E*_g_) ∼ 0.3 eV and low carrier concentration (*n*), Bi_2_Se_3_ displays bipolar effect.^20^ The excitation of minor carriers attenuates the *S*, while the *κ*_e_ is enhanced at the same time, suggesting a small PF and a high *κ*_e_.^21^ Shakouri *et al.* proposed that enlarging the band gap would be an effective method to avoid bipolar conduction.^57^ Increasing *n* directly is another adequate means to minify the effects of minority carriers. In addition, the single valley of conduction band minimum (CBM) (*N*_v_ = 1) will lead to a low DOS and also a small power factor for n-type Bi_2_Se_3_.^22^ In the meantime, suppression of phonon transport is also critical to largely enhance the *zT* of Bi_2_Se_3_. Recently, Zhao *et al.* successfully introduced Sb–Br into the intrinsic semiconductor Bi_2_Se_3_ and obtained a maximum *zT* of 1.4 for n-type BiSb(Se_0.94_Br_0.06_)_3_ at 800 K. That is analogous to the most n-type Te-free TE materials, indicating that Sb–Br codoping is an effective approach in improving TE properties of Bi_2_Se_3_ alloy compounds. They found that Sb doping reduced *κ* on account of strong anharmonicity and soft bonds.^23^ But the electrical transport properties of BiSbSe_3_ is still poor because of the extremely low *n* and the imbalance between the effective mass and *n*. Then, *via* heavy Br-doping in BiSbSe_3_, Liu *et al.* acquired a high *n* and further optimized TE properties. Although there is a computational work about the electronic structure of BiSbSe_3_ alloy^22^ and a parameterized model theoretical analysis about the effects of Br doping,^23^ the detailed physical reasons in atomic level for Br codoping to further significantly improve TE properties have not been investigated yet. Particularly, the relationship between the crystalline atomic structure and the TE performance for Bi_2−*x*_Sb_*x*_(Se_1−*y*_Br_*y*_)_3_ (*x* = 0, 1, 2; *y* = 0, 0.08) is still unclear so far.

In this work, we report a systematic first-principles study on the TE properties of the Bi_2−*x*_Sb_*x*_(Se_1−*y*_Br_*y*_)_3_ (*x* = 0, 1, 2; *y* = 0, 0.08) alloy. The calculations are based on the density functional theory (DFT) for the atomic and electronic structures and based on the Boltzmann transport theory for the electron and phonon transport processes. It has been shown in literature that DFT is one of the most common and successful theoretical tools for probing TE materials, such as Cu_12_Sb_4_S_13_.^56^*x* = 0, 1 in the Bi_2−*x*_Sb_*x*_Se_3_ system is identified to be the transition boundary of the rhombohedral structure and orthorhombic structure. The strong hybridization between Sb and Se atoms in the complex crystal structure of orthorhombic BiSbSe_3_ with alloying 50% Sb on Bi sites makes it have a lower *κ*_L_. In addition, the increase of structural symmetry is helpful to obtain a large energy valley degeneracy (*N*_v_), and a large *σ* can be achieved by increasing conductive channels. The *N*_v_ and chemical bond softening can be further improved significantly by codoping 8% Br at Se sites. The improved *N*_v_ will enhance the 
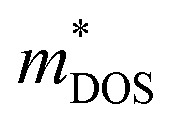
 without reducing the *μ*. The further softened phonons are beneficial to enhancing anharmonic scattering and further decrease the *κ*_L_. The Br codoping also moves the Fermi level (*E*_F_) into the conduction band (CB), resulting in a significant increase of *n* and *σ*. Interestingly, the increase of *n* does not reduce largely *S* because of the increased 
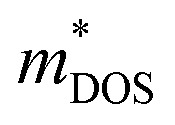
. Eventually, a peak *zT* value of ∼0.96 at 800 K can be realized in n-type alloy compounds BiSb(Se_0.92_Br_0.08_)_3_ with the carrier concentration of 9.22 × 10^19^ cm^−3^.

## Results and discussion

### Crystal structure and electronic structure

**Fig. 1:**
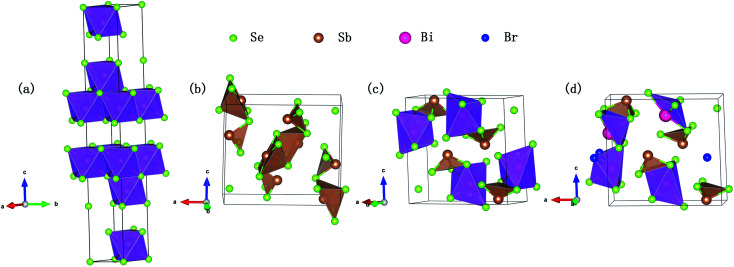
Crystal structure of Bi_2_Se_3_ (a), Sb_2_Se_3_ (b), BiSbSe_3_ (c), and BiSb(Se_0.92_Br_0.08_)_3_ (d), with Bi, Sb, Se, and Br atoms shown as red, brown, green, and blue spheres, respectively.

Bi_2_Se_3_ compound has a rhombohedral crystal structure with space group *D*^5^_3d_(*R*3̄*m*) no. 166 (Fig. 1(a)). In order to generate a reliable atomic structure for this compound, we first optimized its crystal structure with PBE functional as implemented in VASP in the total energy method. Its lattice constants *a*, *b* and *c* are found to be 4.19, 4.19 and 30.8 Å, respectively, which are in good agreement with the experimental values 4.14, 4.14 and 28.63 Å,^27^ proving the reliability of our theoretical modeling. The distinct layered structure consists of a regular octahedron structure with Bi atom as the center and Se atom as the vertex. On the other hand, Sb_2_Se_3_ exhibits an orthorhombic structure composed of a tetragonal conical pentahedron with Sb as the bottom center and Se as the vertex, as shown in Fig. 1(b). According to the previous results of Zhao *et al.*,^23^ the substitution of Sb for 50% Bi atoms in Bi_2_Se_3_ will induce a phase transition from the rhombohedral structure to the orthorhombic structure. Therefore, we utilize Bi atoms to replace the two nonequivalent sites of Sb1 atom in Sb_2_Se_3_, and adopt a structure with the lower energy to simulate the BiSbSe_3_. It displays a significant chain-like structure of orthorhombic phase by forming an octahedral structure with Bi atom as the center and Se atom as the vertex, and a triangular pyramidal tetrahedron with Sb and three Se atoms as the vertex is also formed (Fig. 1(c)).

This complex crystal structure of BiSbSe_3_ is favorable to reducing the *κ*_L_ because of the structural phase transition and the formation of the chain-like structure. Since the structural rhombohedral–orthorhombic phase transition is related to the breakage of part of the cation–anion bonds, half of the octahedral coordination cations are converted to five coordination, as reported previously by Yang *et al.*^22^ Finally, we consider the possible substitution of single Br atom for Se. There are three nonequivalent sites of Se atom in BiSbSe_3_, therefore, three possible structures can be formed. It is found by calculation that the total energies per cell of the three structures are −78.373, −78.285 and −78.308 eV, respectively. Furthermore, we calculate the energy band structures and thermoelectric transport properties for the three structures, respectively. The results are compared in Fig. S1 and S2,† which show that the results are qualitatively similar. Herein, we take the lowest energy structure with Se3 replaced by Br atom to model the BiSb(Se_0.92_Br_0.08_)_3_.

As shown in Fig. 1(d), this structure also possesses a stable orthorhombic structure (space group *Pnma*) but is more complex: not only an octahedral structure with Bi atom as the center and Se atom as the apex, and a triangular pyramidal tetrahedron with one Sb and three Se atoms as the apex are formed, but also formed is an octahedral structure with Bi as the center and both Se and Br atoms as the vertices. This more complex structure may be helpful to further suppressing the lattice thermal conductivity.

**Fig. 2:**
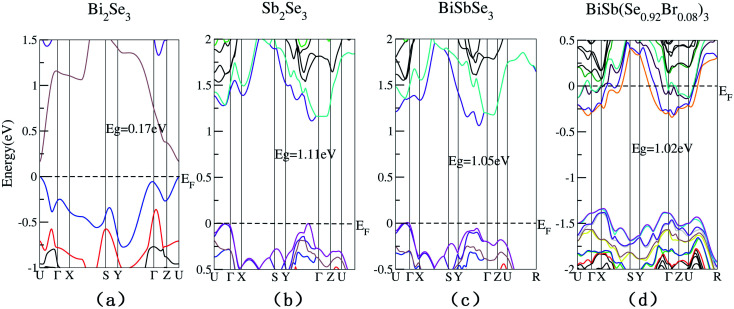
Calculated electronic band structures of Bi_2_Se_3_ (a), Sb_2_Se_3_ (b), BiSbSe_3_ (c), and BiSb(Se_0.92_Br_0.08_)_3_ (d).

The conduction band and valence band model can be used to analyze how the carrier concentration and the band gap affect the TE properties. The calculated electronic band structures of Bi_2−*x*_Sb_*x*_(Se_1−*y*_Br_*y*_)_3_ (*x* = 0, 1, 2; *y* = 0, 0.08) are shown in Fig. 2. It is worth mentioning that the band structures of Bi_2−*x*_Sb_*x*_(Se_1−*y*_Br_*y*_)_3_ (*x* = 1, 2; *y* = 0, 0.08) are given by the TB-mBJ functional, while those of Bi_2_Se_3_ (band gap ∼ 0.17 eV) is given by the PBE-GGA functional so that we can have a direct comparison with a previous PBE-GGA result (∼0.16 eV), as shown in Table 1.

**Table 1:** Lattice parameters and band gaps of Bi_2−*x*_Sb_*x*_(Se_1−*y*_Br_*y*_)_3_ (*x* = 0, 1, 2; *y* = 0, 0.08)

Compounds	Experimental value				Previously calculated value				Our calculated value			
	*a*	*b*	*c*	*E*_g_	*a*	*b*	*c*	*E*_g_	*a*	*b*	*c*	*E*_g_
Bi_2_Se_3_	4.14^a^	4.14^a^	28.63^a^	0.25^a^	4.17^a^	4.17^a^	30.29^a^	0.16^a^	4.19	4.19	30.80	0.17
Sb_2_Se_3_	11.77^b^	3.96^b^	11.62^b^	1.17^b^	11.53^b^	3.96^b^	11.22^b^	0.75^b^	12.82	4.03	11.53	1.11
BiSbSe_3_	11.70^c^	3.91^c^	11.62^c^	0.88^c^	12.59^c^	4.10^c^	11.65^c^	0.63^c^	12.63	4.10	11.62	1.05
BiSb(Se_0.92_Br_0.08_)_3_	10.52^d^	5.26^d^	10.17^d^	—	11.84^d^	4.07^d^	11.67^d^	—	12.36	4.16	11.65	1.02

a Result from ref. 27.

b Result from ref. 28.

c Result from ref. 29.

d Result from ref. 23.

One can see in Table 1 that the previous PBE-GGA result of the *E*_g_ of Sb_2_Se_3_ (∼0.7 eV) is largely underestimated with respect to the experimental value (∼1.17 eV), while our TB-mBJ result (∼1.11 eV) is in good agreement. In order to ensure the accuracy of the calculated results, we use TB-mBJ to calculate the band structures of Bi_2−*x*_Sb_*x*_(Se_1−*y*_Br_*y*_)_3_ (*x* = 1, 2; *y* = 0, 0.08). The SOC are considered in our calculations due to the heavy element. As can be seen in Table 1 and Fig. 2, the Bi_2−*x*_Sb_*x*_(Se_1−*y*_Br_*y*_)_3_ (*x* = 1, 2; *y* = 0, 0.08) have much larger band gaps than Bi_2_Se_3_, which can largely suppress the intrinsic excitation and the bipolar effect to prevent them from reaching a higher *zT* value. In addition, the 8% Br codoping further makes the Fermi level (*E*_F_) shifted to deeper conduction states, indicating that the Br codoping increases significantly the majority carrier concentration.

**Fig. 3:**
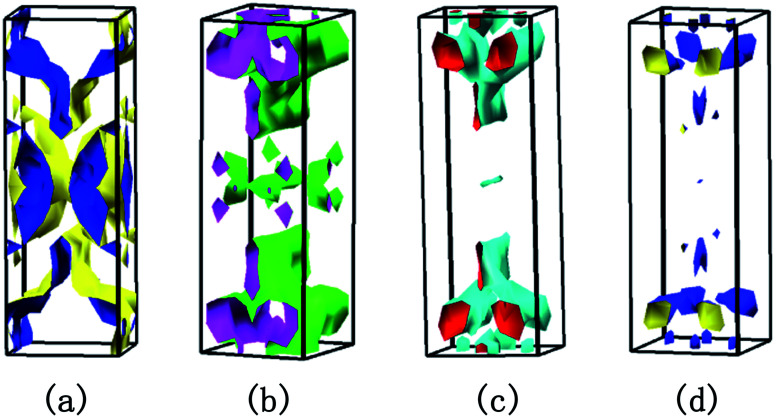
The energy isosurfaces at 0.37 eV of BiSb(Se_0.92_Br_0.08_)_3_ (space group: *Pnma*, no. 62). (a), (b), (c) and (d) are the four conduction bands traversed by Fermi level from bottom to top, respectively.

As is well known, a large *N*_v_ is beneficial to a large DOS effective mass 
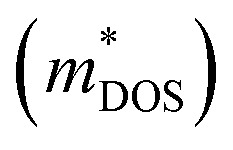
 without deterioration of the carrier mobility *μ*.^4,39^*N*_v_ is the effective total number of independent carrier pockets or valleys in the Brillouin zone, including both symmetry and orbital degeneracies. For Bi_2_Se_3_, it can be seen that alloying 50% Sb on Bi sites increases the valley number of effective energy from the degeneracy 2 to 10. As one can see in Fig. 2(c), the energy difference between the CBM and the fourth valence valley is less than ∼0.14 eV, which is smaller than the 0.15 eV between the first and the second valence bands of PbTe.^40^ Such small energy difference can be easily crossed at elevated temperatures, making it possible to improve the electrical transport properties. Furthermore, 8% Br subsequent codoping at Se12 position exhibits remarkably distinct electronic structures, which further significantly increases the effective *N*_v_, accompanied by pushing the *E*_F_ deep into the band structure, which significantly enlarges the *n*. A high *N*_v_ number generally results in a larger 
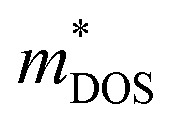
 for the conduction band and eventually maintains a high *S* for n-type samples in spite of the increased *n*. Interestingly, the conduction bands of BiSb(Se_0.92_Br_0.08_)_3_ are much more complex than those of BiSbSe_3_: the activated multiple conduction-band minima lie close together in energy, forming complex multiband valence states. Another illustration of the complex band structure is shown in the Fermi surface, which has multiple types of valleys coming from the four conduction bands of *E*_F_ crossing, all within a small energy window (see Fig. 3(a)–(d)). Such a good band feature may be associated with excellent thermoelectric properties, as found in other thermoelectric materials.^39,41^

**Fig. 4:**
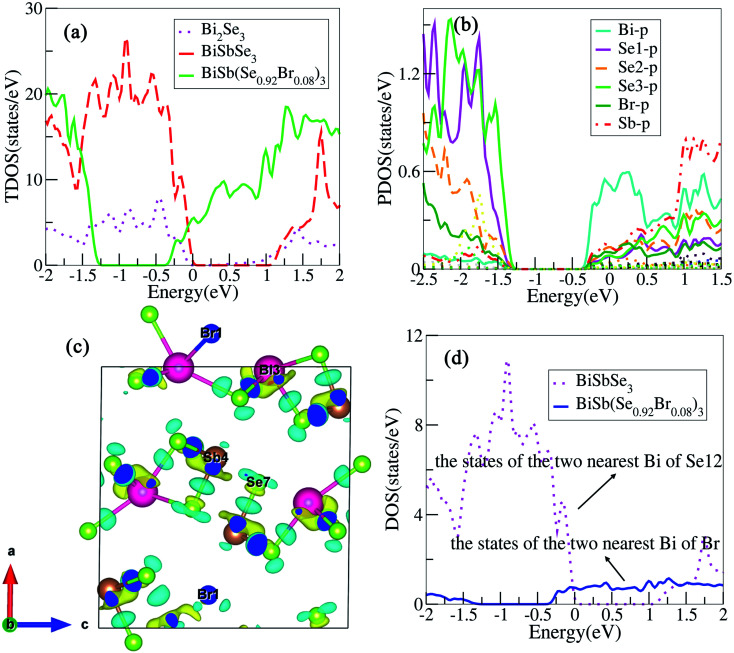
(a) Calculated the TDOS of Bi_2_Se_3_, BiSbSe_3_, and BiSb(Se_0.92_Br_0.08_)_3_. (b) and (c) are the PDOS, the charge density difference of BiSb(Se_0.92_Br_0.08_)_3_, respectively. The colors yellow and cyan in (c) represent the negative and positive charge differences, respectively. (d) Projected density of states on the two nearest Bi atoms of Se12 and Br.

To clearly understand the states near the *E*_F_, we calculate the total density of states (TDOS) for Bi_2_Se_3_, BiSbSe_3_, and BiSb(Se_0.92_Br_0.08_)_3_ respectively, as shown in Fig. 4(a). It should be noted that TDOS near the Fermi level of doped Bi_2_Se_3_ is larger than that of pristine Bi_2_Se_3_. 50% Sb doping on Bi sites of Bi_2_Se_3_ slightly increased the TDOS near the *E*_F_. This is mainly originated from the substitution of Sb for Bi, which increases *N*_v_. Furthermore, 8% Br codoping at Se12 site in BiSbSe_3_ tremendously increases the TDOS in the vicinity of the *E*_F_, which will enhance *σ* significantly. These results manifest that Sb and Br dual doped Bi_2_Se_3_ can be expected to have enhanced electrical transport and thermoelectric properties. To analyze the reasons for the enhanced TDOS by Br substitution, we calculate the partial density of states (PDOS) of BiSb(Se_0.92_Br_0.08_)_3_. As can be seen in Fig. 4(b), the bottom of CB edge is mainly contributed by the hybridized p orbitals from Bi, Se, Br, and Sb atoms (see the next section for more details). Thus, the Br atom replacement on the Se12 site can effectively adjust the band structure near the CB edge, and strongly enhances the thermoelectric performance.

### Bonding properties

To visualize the electronic environments and bonding conditions, charge density difference (CDD) is calculated for BiSb(Se_0.92_Br_0.08_)_3_ compound. Fig. 4(c) reveals that the negative electron density is mainly around atoms Bi and Sb, meaning overall Bi and Sb atoms donate their valence electrons to the [Se_0.92_Br_0.08_]_3_^2−^. The electronegativity on the Pauling scale for Bi, Sb, Se and Br are 2.02, 2.05, 2.55, and 2.96, respectively. The electropositive elements Bi and Sb dedicate all of their valence electrons to the more electronegative elements Se and Br, ultimately, BiSb(Se_0.92_Br_0.08_)_3_ can be described as (BiSb)^3+^(Se_0.92_Br_0.08_)_3_^2−^. It coincides with the above analysis that, in BiSb(Se_0.92_Br_0.08_)_3_, Bi and Sb atoms tend to lose electrons, while Se and Br atoms tend to gain electrons owing to their larger electronegativity. These also manifest that Br–Bi has a stronger interaction than Se–Bi. In fact, the stronger hybridization between Br and Bi atoms will conduce to increasing the DOS near the CB edge, as indicated in Fig. 4(d). The electrons between the Bi and Br atoms mainly localizes around Br, while a certain number of electrons appear around the center position between Bi and Se, and Sb and Se atoms, indicating Bi and Br atoms incline to form an ionic bond, while there is a distinct covalent bond characteristics between Bi and Se, and Sb and Se atoms. The ionicity of the bonding in a material usually is characterized by the electronegativity difference (Δ*χ*). The larger Δ*χ* (≈0.9 between Br and Bi atoms), the more likely there will be interaction between carriers and the optical vibrations of the lattice atoms,^42^ thus suppressing the *κ*_L_. For instance, observations by Ioffe *et al.* showed a decrease of *κ* with an increase of the ionicity of the bonds between atoms.^42^ Spitzer reported a correlation between increasing coordination number (CN) in a crystal structure and decreasing *κ*_L_ (by relating the *κ*_L_ to the strength of the chemical bonds in a material).^42^ In our case, the bond lengths between atoms after Br codoping become longer, namely, Se12–Bi bond lengths (2.81, 3.12 Å) in the BiSbSe_3_ are shorter than the corresponding Br–Bi bond lengths (3.05, 3.23 Å) in BiSb(Se_0.92_Br_0.08_)_3_. This implies that the Br–Bi bond could be relatively weaker according to the bonding descriptor contact developed by Deringer *et al.*^28^ The weaker chemical bond among Br and Bi atoms will be also a factor leading to the low *κ*_L_ (see the next section), as also recognized recently in α-MgAgSb.^43^ In addition, the big “mushroom” CDD shape around Bi/Sb is a clear indicator of the existence of lone-pair electrons, which is alike to the case of CuSbS_2_.^44^ According to the valence shell electron pair repulsion (VSEPR) theory,^45^ the lone-pair electrons almost occupy one bonding site. Hence, the unsymmetrical and disorderly coordinated environment of bonds in BiSb(Se_0.92_Br_0.08_)_3_ indicates the possibility of strong anharmonicity by significantly enhancing heat carrying phonon scatterings. This will also result in ultralow *κ*_L_.

### Low lattice thermal conductivities

**Fig. 5:**
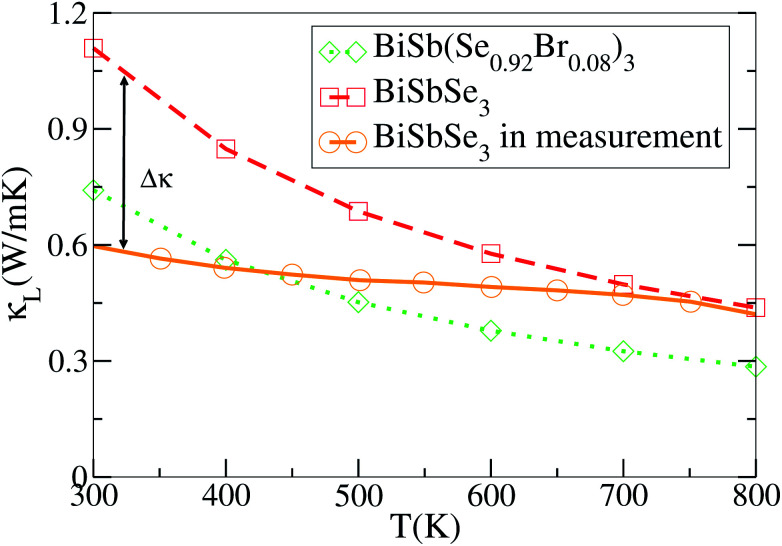
Experimental and DFT-calculated lattice thermal conductivities as functions of temperature.

To achieve a high thermoelectric figure of merit, a low lattice thermal conductivity is essential. Therefore, insights into the origin of the extremely low thermal conductivity is meaningful for exploiting high performance thermoelectric materials. Previous work predicted that BiSbSe_3_ could possess a low *κ*_L_.^23^ Nevertheless, the prediction was based on a strong dependence of the elastic properties on volume, and the resulting large Grüneisen parameter (*γ*, an indicator of anharmonicity) suggests a strong anharmonicity. In this work, we precisely calculate the *κ*_L_ as a function of temperature for BiSbSe_3_ and BiSb(Se_0.92_Br_0.08_)_3_ using the full linearized ShengBTE code combined with DFT, which is well known for quantitative predictive power.^46^ The results are presented in Fig. 5.

As mentioned previously, the substitution of Sb for 50% Bi in Bi_2_Se_3_ decreases the *κ*_L_ partly due to the structural phase transition from rhombohedral to orthorhombic, and partly due to the formation of the more complex chain-like structure with respect to the layered structure, which induces enhanced intrinsic phonon scatterings. In Fig. 5 our *ab initio* results are compared with the experimental *κ*_L_ values of polycrystalline samples cited from ref. 23. It is worth mentioning that the calculated values are averaged over the three principal axes for the purpose of better vision and comparison with the experimental measurements on polycrystalline samples. It can be seen that the predicted values of BiSbSe_3_ are below 1.2 W m^−1^ K^−1^ at 300 K, which is pretty low in thermoelectric materials.^47,48^ However, the experimental values are even much lower at low temperatures though the agreement between theory and experiment is improved at high temperatures. The large discrepancy in *κ*_L_ at low temperatures may not be entirely attributed to grain boundary scattering, since this effect is usually small in materials with intrinsically low *κ*_L_, where heat carrying phonons have smaller mean free paths than the size of the grains.^49,50^ It has to be noted that the theoretical calculation does not take into account the phonon scattering due to dynamic disorder, an omission that may be responsible for the overestimation of *κ*_L_.

Here a possible mechanism is that the underlying atomic disorder plays an appreciable role in reducing *κ*_L_ at relatively low temperatures. While at high temperatures atoms occupy higher-symmetry positions,^51^ and intrinsic phonon–phonon scattering then dominates, as described by Eivari *et al.*^55^ For example, the previously mentioned lone-pair electrons may provide an origination of thus disorder which can act as a phonon-blocking mechanism that may help facilitate an ultralow *κ*_L_. Moreover, we find that 8% Br codoping is also effective to further reduce *κ*_L_ as shown in Fig. 5. BiSb(Se_0.92_Br_0.08_)_3_ shows a lower *κ*_L_ than that of BiSbSe_3_ at the entire temperature. For instance, their *κ*_L_ are 0.29 and 0.44 W m^−1^ K^−1^ at 800 K, respectively. Clearly, the chemical bond softening between Br and Bi atoms is considerably higher than that between Se12 and Bi. As we know, the strong anharmonicity usually not only relies directly on the unsymmetrical and chemical bonds between the atoms in the crystal but also is often associated with atoms and their near neighbors (large coordination numbers). The calculated *κ*_L_ of BiSb(Se_0.92_Br_0.08_)_3_ is found to be in good agreement with an available experimental value 0.22 W m^−1^ K^−1^ at 800 K.^23^

### Promising electrical transport properties

**Fig. 6:**
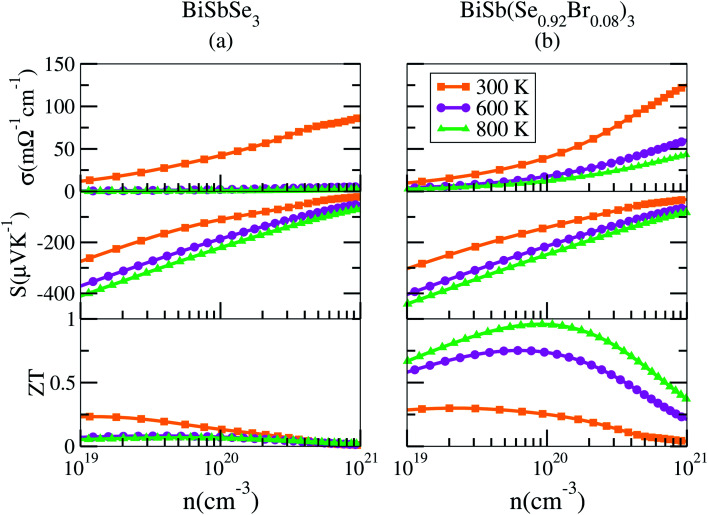
Calculated transport properties of n-type BiSbSe_3_ (a), and BiSb(Se_0.92_Br_0.08_)_3_ (b), respectively.

To shed light on the influences of Sb–Br codoping, herein the electrical transport properties of BiSbSe_3_ and BiSb(Se_0.92_Br_0.08_)_3_ are calculated as functions of carrier concentration (*n*) at 300 K, 600 K, and 800 K within the framework of the semiclassical Boltzmann transport theory.^52^ While it is possible to calculate *σ*/*τ* as a function of *n* and *T*, but it is not possible to calculate *σ* itself without the scattering rate *τ*^−1^. Here, the strategy previously used by Ong *et al.*^53^ is adopted with available experimental data^23^ to estimate the relaxation time *τ* = *C*_0_*T*^−1^*n*^−1/3^ with *τ* in s, *T* in K, and *n* in cm^−3^. The specific details are that we used 800 °C data from Liu and coworkers,^23^ who made measurements on the material. They report a thermopower *S* = −202.462 μV K^−1^ at this temperature. By comparing with the calculated *S*(*T*, *n*), we obtain a value *n* = 1.77 × 10^20^ cm^−3^ for this sample. The reported experimental *σ* is 172.899 S cm^−1^, which combined with the calculated *σ*/*τ* yields *τ* = 2.345 × 10^−15^ s for this particular sample at 800 °C. As mentioned, we then calculate *σ* as *σ*/*τ* × *τ*. Ultimately, the results for BiSbSe_3_ and BiSb(Se_0.92_Br_0.08_)_3_ are plotted in Fig. 6.

Based on the estimated *τ*, we can calculate the *κ*_e_ using the BoltzTraP2 code. Unfortunately, with the significant increase of *n*, the *κ*_e_ augments from ∼0.05 W m^−1^ K^−1^ of BiSbSe_3_ at 300 K, to ∼0.34 W m^−1^ K^−1^ of BiSb(Se_0.92_Br_0.08_)_3_ at 800 K. However, as discussed previously, Br codoping notably reduces the *κ*_L_ from ∼1.11 W m^−1^ K^−1^ at 300 K to ∼0.29 W m^−1^ K^−1^ at 800 K. Therefore, a favorable *κ* (∼0.63 W m^−1^ K^−1^) could still be obtained for BiSb(Se_0.092_Br_0.08_)_3_ at 800 K. Then, by combining the calculated *κ*_L_, *κ*_e_, *S*, and *σ* of BiSbSe_3_ and BiSb(Se_0.92_Br_0.08_)_3_, we determine their *zT*s at different temperatures and carrier concentrations. Fascinatingly, compared with 50% Sb doped BiSbSe_3_ (maximum *zT* ∼ 0.23 with the optimal *n* of 1.163 × 10^19^ cm^−3^ at 800 K, see Fig. 6(a)), further 8% Br codoping enhances *σ*, hence strikingly improves the *zT* (maximum ∼ 0.96 with the optimal *n* of 9.224 × 10^19^ cm^−3^ at 800 K, see Fig. 6(b)). BiSb(Se_0.92_Br_0.08_)_3_ exhibits a still high *S* that may originate from the increase of 
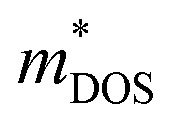
 owing to the increase of *N*_v_ through Br codoping. On the other hand, the remarkably enhanced *σ* shown in Fig. 6(b) may originate from the increases of *n* and/or *μ*.

**Fig. 7:**
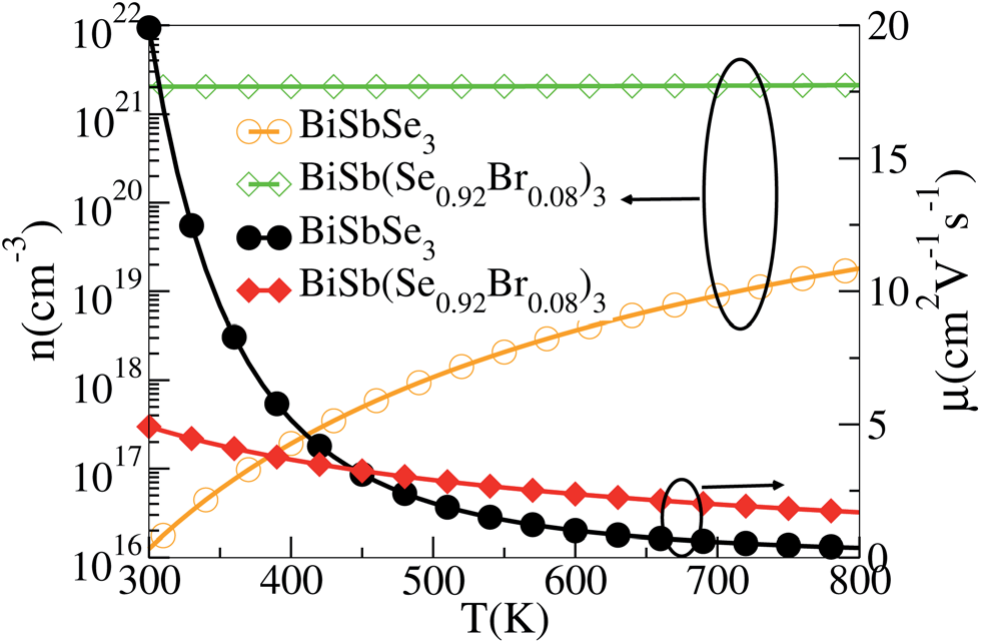
Calculated *n* and *μ* of BiSb(Se_0.92_Br_0.08_)_3_ as functions of temperature.

To show this more clearly, we calculate *n* and *μ* as functions of temperature for BiSbSe_3_ and BiSb(Se_0.92_Br_0.08_)_3_, as depicted in Fig. 7. *μ* is obtained from the calculated *σ* and *n* by using the expression *μ* = *σ*/*ne*. One can see that, 8% Br codoping dramatically enlarges *n*, and in the meantime, *μ* is still promoted at medium high temperatures. The significant increase of *n* in BiSb(Se_0.92_Br_0.08_)_3_ is essentially due to the one more electron of Br atom with respect to Se. Thus, Br atom acts as an electron donor though it has a large electronegativity. Consequently, the *E*_F_ shifts deeper in the CB and the differential conductivity turns into more symmetric regarding the Fermi level.^54^ Our Bader charge analyses show consistently that each nearest neighbor Bi atom of Br averagely obtains more 0.44 electrons from the Br atom than that from Se. In addition, the hybridization of the electronic states then provides a large DOS around the Fermi level, as already shown in Fig. 4(d). A direct result is the remarkably enhanced *σ*. However, the dramatically increased *n* in the Sb–Br codoping system does not significantly reduce the *S*: it is −261.93 μV K^−1^ for BiSbSe_3_ at 300 K and −254.12 μV K^−1^ for BiSb(Se_0.92_Br_0.08_)_3_ at 800 K. Based on the aforementioned data and analyses, this explicit increase is most likely due to the *N*_v_ augment, which is the key parameter for making a compromise between *n* and *S*. Normally, the optimum *n* for most good TE materials is in the range of 10^19^ to 10^21^ cm^−3^. Optimization of the carrier concentration is still one of the most effective approaches for improving TE performance. Especially, in the case of some TE materials with intrinsically low thermal conductivity, a high *zT* can be obtained solely through doping.

## Methods

### First-principles calculations

The atomic structures of Bi_2−*x*_Sb_*x*_(Se_1−*y*_Br_*y*_)_3_ (*x* = 0, 1, 2; *y* = 0, 0.08) are optimized utilizing the plane-wave projector augmented wave (PAW) method^24^ as implemented in the Vienna *ab initio* simulation package (VASP)^25^ based on DFT. The Perdew–Burke–Ernzerhof parametrization of the generalized gradient approximation (PBE-GGA) is used for the exchange–correlation potential. The wave function is expanded using plane waves with a kinetic energy cutoff of 400 eV. A (5 × 15 × 5) Monkhorst–Pack^26^*k*-point mesh is used for the Brillouin zone (BZ) sampling. The spin–orbit coupling (SOC) is considered in all the calculations due to the heavy element. The geometry optimization is performed for every Br doping configuration by relaxing both atomic positions and lattice constants. The energy convergence criterion is chosen to be 10^−7^ eV. The Hellmann–Feynman forces on each ion are less than 0.001 eV Å^−1^. The residual stress is set to be less than 0.1 GPa. Here, we discuss only the configuration with the lowest total energy. The calculated lattice constants are listed in Table 1. These values agree well with previous results reported in literature.^23,27–29^

The electronic structures of Bi_2−*x*_Sb_*x*_(Se_1−*y*_Br_*y*_)_3_ (*x* = 0, 1, 2; *y* = 0, 0.08) are then calculated using the linearized augmented plane wave (LAPW) method,^30^ as implemented in the WIEN2k code.^31^ The muffin-tin radii (RMT) are set to 2.5, 2.46, 2.46, and 2.5 a.u. for Bi, Sb, Se, and Br, respectively. The cutoff parameter RMT × *K*_max_ = 9 (*K*_max_ is the magnitude of the largest *k* vector) is used. The self-consistent DFT calculations are performed with a (5 × 15 × 5) *k*-point mesh in the irreducible BZ, and the total energy is converged to within 0.0001 Ry. Since the local or semilocal exchange–correlation approximation underestimates band gaps as the presence of artificial self-interaction and the absence of the derivative discontinuity in the exchange–correlation functional,^32^ band gaps with better accuracy are calculated using the Tran–Blaha modified Becke–Johnson (TB-mBJ) functional.^33^ The calculated electronic structures with the TB-mBJ functional are further used to obtain electrical transport properties. The transport calculations are carried out using the semiclassical Boltzmann theory, as implemented in the BoltzTraP code^34^ within the constant scattering time approximation, by taking 20 000 *k* points in the irreducible BZ.

The lattice thermal conductivity can be determined by using an iterative self-consistent method for solving the phonon Boltzmann transport equation as implemented in the ShengBTE code.^35,36^ The harmonic lattice dynamics and the second-order interatomic force constants (IFCs) are determined by the density functional perturbation theory (DFPT)^37^ implemented in VASP with a supercell of 1 × 3 × 1 and the PHONOPY code^38^ interfaced to VASP. The third-order anharmonic IFCs are extracted from the DFT calculations by applying the finite displacement method with a supercell of 1 × 3 × 1 and a truncation for next–nearest–neighbor interactions up to the tenth order. As for the *k*-point sampling of the BZ, our test calculations adopting a 1 × 3 × 1 and a 3 × 3 × 3 *k*-point mesh show that the thermal conductivities are converged very well. In this work, a 3 × 3 × 3 mesh is used for all the supercell calculations.

## Summary and conclusion

In this work, we performed comprehensive investigations of both the thermal and electrical transport properties of BiSbSe_3_ and BiSb(Se_0.92_Br_0.08_)_3_ by using first-principles calculations combined with the Boltzmann transport theory. The results demonstrate that the dual doping of Sb–Br in Bi_2_Se_3_ leads to both low *κ*_L_ and promising electrical transport properties. First, the chemical bond softening between Br and Bi atoms and lone-pair electrons act as a phonon-blocking mechanism which greatly suppresses *κ*_L_ with strong anharmonicity. Second, the one more electron of Br atom with respect to Se makes the Br atom acts as an electron donor, which dramatically increases the carrier concentration *n* in BiSb(Se_0.92_Br_0.08_)_3_. As a result, the DOS near the *E*_F_ is significantly increased and more conduction bands participate in the electron transport, leading to a remarkably enhanced *σ*. Third, the 8% Br subsequent codoping exhibits remarkably distinct electronic structures and transport properties, which further significantly increases the effective *N*_v_. This multiple degeneracy of conduction band edges is a distinctive feature of BiSb(Se_0.92_Br_0.08_)_3_, enabling an extraordinary high PF by giving rise to a large 
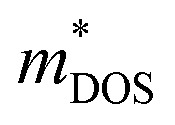
 and thus preserving a large *S* in spite of the dramatically increased *n*. Combined with the low *κ* and excellent electrical transport properties, the n-type BiSb(Se_0.92_Br_0.08_)_3_ presents an outstanding thermoelectric performance, especially around 800 K: the maximum *zT* can reach 0.96 with the optimal *n* of 9.224 × 10^19^ cm^−3^. This work shows theoretically the possibility for crystalline materials to achieve a high thermoelectric performance without introducing defects and/or nanostructures, and provides a possible guidance and inspiration for seeking new promising Te-free thermoelectric materials.

## Data availability

The data that support the findings of this study are available from corresponding author upon reasonable request.

## Author contributions

S.-H. Ke conceived the idea. J. Zhang conducted the simulation and analysis. S. Zhong provided advices on this work. All authors participated in the writing and correction of the manuscript.

## Conflicts of interest

The authors declare no competing interests.

## Supplementary Material

RA-012-D1RA08726F-s001
