# Supplementary material for: Enhanced thermoelectric performance in Sb–Br codoped Bi2Se3 with complex electronic structure and chemical bond softening
Source: RSC Adv. 2022 Jan 11;12(3):1653–62. doi: 10.1039/d1ra08726f (PMC8978978; doi:10.1039/d1ra08726f)
Supplement: RA-012-D1RA08726F-s001 [file RA-012-D1RA08726F-s001.pdf]

Electronic Supplementary Material (ESI) for RSC Advances.
This journal is © The Royal Society of Chemistry 2021

Supporting Information

Enhanced thermoelectric performance in Sb-Br codoped Bi_2Se_3 with complex electronic structure and chemical bond softening

Ju Zhang, Shiqi Zhong, and San-Huang Ke*

MOE Key Laboratory of Microstructured Materials, School of Physics Science and Engineering, Tongji University, 1239 Siping Road, Shanghai 200092, China

E-mail: shke@tongji.edu.cn

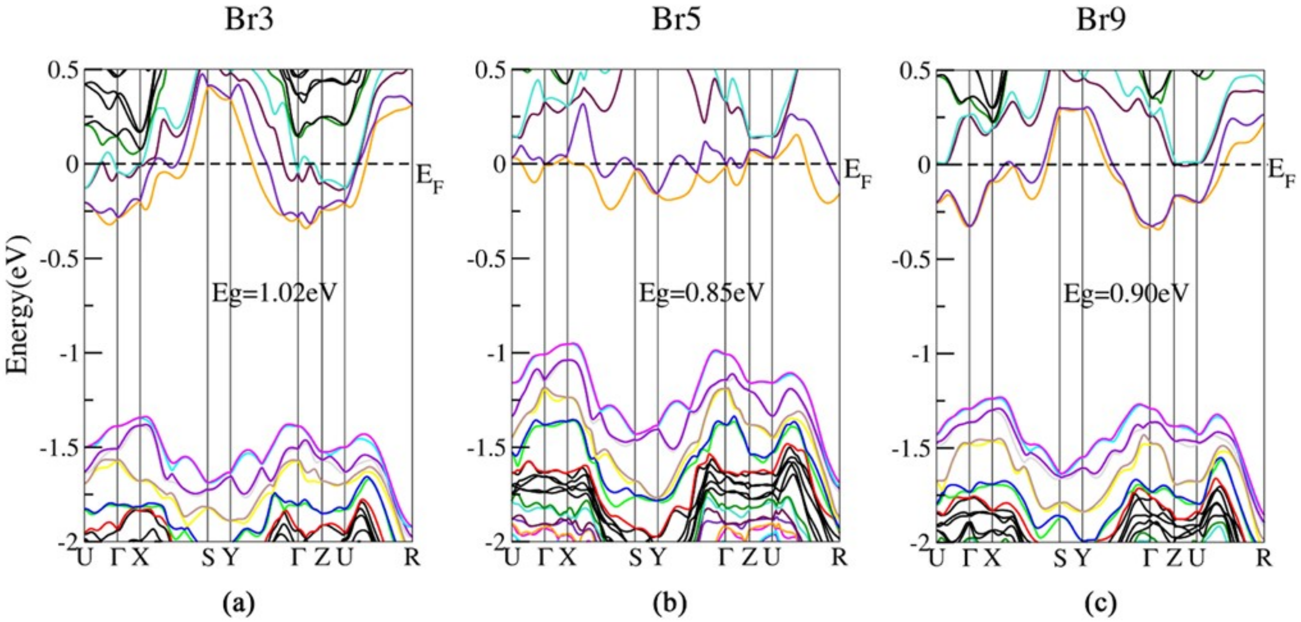

FIG. S1. Calculated electronic band structures of $BiSb(Se_{0.92}Br_{0.08})_3$ with Br doped at three nonequivalent sites.

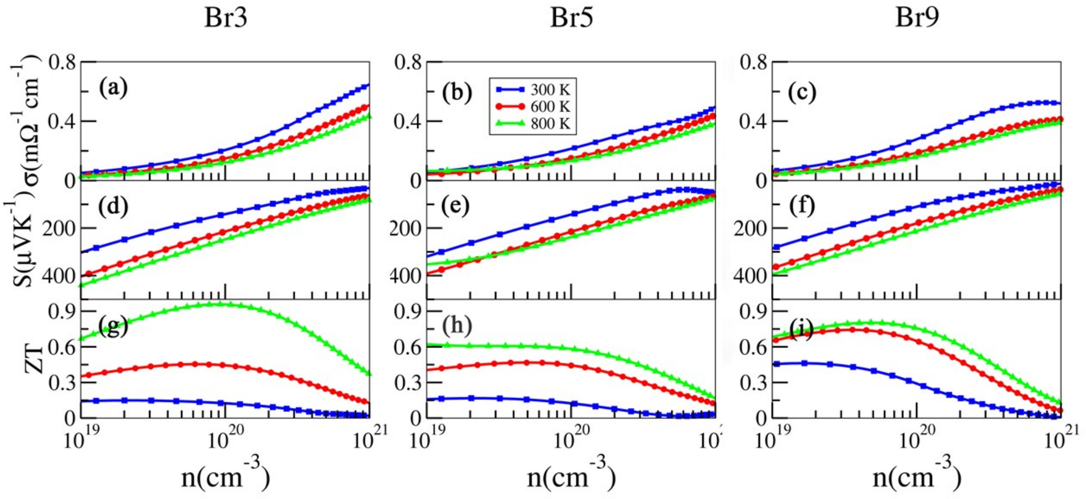

FIG. S2 Calculated transport properties of n-type $BiSb(Se_{0.92}Br_{0.08})_3$ with Br doped at three nonequivalent sites, respectively.

Calculation method: First-principles calculations within the density functional theory (DFT) have been performed using the projector-augmented wave (PAW) method [1], as implemented in the Vienna Ab-initio Simulation Package (VASP) [2]. The exchange-correlation is treated in the generalized gradient approximation (GGA) in the version of Perdew-Burke-Ernzerhof (PBE) [3]. The cutoff energy is set to be 500 eV for all calculations. A Monkhorst-Pack Γ-centered 5 ×15 ×5 k mesh is used for the Brillouin zone sampling. The atomic structures, including lattice parameters and atomic positions, are fully relaxed until the maximum residual ionic force is below 0.001 eV/Å, and the total energy difference is converged to within 10^{-8} eV. The spin-orbit coupling (SOC) are considered in our calculations due to the heavy element.

References

[1] P. E. Blöchl, Projector Augmented-Wave Method. Phys. Rev. B 50, 17953 (1994).

[2] G. Kresse and J. Furthmuller, Efficiency of Ab-Initio Total Energy Calculations for Metals and Semiconductors Using a Plane-Wave Basis Set. Comput. Mater. Sci. 6, 15 (1996).

[3] H. J. Monkhorst and J. D. Pack, Special Points for Brillouin-Zone Integrations. Phys. Rev. B 13, 5188 (1976).
